# Do common infections trigger disease-onset or -severity in CTLA-4 insufficiency?

**DOI:** 10.3389/fimmu.2022.1011646

**Published:** 2022-11-02

**Authors:** Máté Krausz, Noriko Mitsuiki, Valeria Falcone, Johanna Komp, Sara Posadas-Cantera, Hanns-Martin Lorenz, Jiri Litzman, Daniel Wolff, Maria Kanariou, Anita Heinkele, Carsten Speckmann, Georg Häcker, Hartmut Hengel, Laura Gámez-Díaz, Bodo Grimbacher

**Affiliations:** ^1^ Institute for Immunodeficiency, Medical Center – University of Freiburg, Faculty of Medicine, University of Freiburg, Freiburg, Germany; ^2^ Center for Chronic Immunodeficiency (CCI), Medical Center – University of Freiburg, Faculty of Medicine, University of Freiburg, Freiburg, Germany; ^3^ Department of Rheumatology and Clinical Immunology, Medical Center - University of Freiburg, Faculty of Medicine, University of Freiburg, Freiburg, Germany; ^4^ Faculty of Biology, University of Freiburg, Freiburg, Germany; ^5^ Institute of Virology, University Medical Center and Faculty of Medicine, University of Freiburg, Freiburg, Germany; ^6^ Institute of Medical Microbiology and Hygiene, Medical Center - University of Freiburg, Faculty of Medicine, University of Freiburg, Freiburg, Germany; ^7^ Division of Rheumatology, Department of Internal Medicine V, University of Heidelberg, Heidelberg, Germany; ^8^ Department of Clinical Immunology and Allergology, St. Anne’s University Hospital in Brno and Medical Faculty, Masaryk University, Brno, Czechia; ^9^ Department of Internal Medicine III, University Hospital Regensburg, Regensburg, Germany; ^10^ Department of Immunology and Histocompatibility, Centre for Primary Immunodeficiencies, “Aghia Sophia” Children’s Hospital, Athens, Greece; ^11^ Center for Pediatric Rheumatology, Olgahospital, Stuttgart, Germany; ^12^ Department of Pediatric Hematology and Oncology, Center for Pediatrics and Adolescent Medicine, Faculty of Medicine, University of Freiburg, Freiburg, Germany; ^13^ DZIF – German Center for Infection Research, Satellite Center Freiburg, Freiburg, Germany; ^14^ CIBSS – Centre for Integrative Biological Signalling Studies, University of Freiburg, Freiburg, Germany; ^15^ RESIST – Cluster of Excellence 2155 to Hannover Medical School, Satellite Center Freiburg, Freiburg, Germany

**Keywords:** cytotoxic T-lymphocyte antigen 4 (CTLA-4), immunodeficencies, immune dysregulation, inborn errors of immunity (IEI), disease modifiers

## Abstract

**Purpose:**

Heterozygous mutations in *CTLA4* lead to an inborn error of immunity characterized by immune dysregulation and immunodeficiency, known as CTLA-4 insufficiency. Cohort studies on *CTLA4* mutation carriers showed a reduced penetrance (around 70%) and variable disease expressivity, suggesting the presence of modifying factors. It is well studied that infections can trigger autoimmunity in humans, especially in combination with a genetic predisposition.

**Methods:**

To investigate whether specific infections or the presence of specific persisting pathogens are associated with disease onset or severity in CTLA-4 insufficiency, we have examined the humoral immune response in 13 *CTLA4* mutation carriers, seven without clinical manifestation and six with autoimmune manifestations, but without immunoglobulin replacement therapy against cytomegalovirus (CMV), Epstein-Barr virus (EBV), herpes simplex virus 1/2 (HSV 1/2), parvovirus B19 and *Toxoplasma gondii*. Additionally, we have measured FcγRIII/CD16A activation by EBV-specific IgG antibodies to examine the functional capabilities of immunoglobulins produced by *CTLA4* mutation carriers.

**Results:**

The seroprevalence between affected and unaffected *CTLA4* mutation carriers did not differ significantly for the examined pathogens. Additionally, we show here that *CTLA4* mutation carriers produce EBV-specific IgG, which are unimpaired in activating FcγRIII/CD16A.

**Conclusions:**

Our results show that the investigated pathogens are very unlikely to trigger the disease onset in CTLA-4-insufficient individuals, and their prevalence is not correlated with disease severity or expressivity.

## Introduction

Cytotoxic T-lymphocyte antigen 4 (CTLA-4) is a negative immune regulator constitutively expressed on regulatory T cells (Treg) and upregulated on activated T cells. CTLA-4 insufficiency in humans was first reported in 2014 as a monogenetic disorder caused by heterozygous germline mutations in *CTLA4* (1, 2). Aberrant CTLA-4 function leads to uncontrolled T cell responses that clinically manifests as an immune dysregulation syndrome, characterized by autoimmunity and paradoxically by immunodeficiency. Particularly, clinical signs and symptoms of CTLA-4 insufficiency include hypogammaglobulinemia, autoimmune cytopenia, lymphoproliferation (such as lymphadenopathy or splenomegaly), enteropathy, neurological or skin involvement. Interestingly, multiple cohort studies report an incomplete penetrance of around 70% (3–5), suggesting that additional factors might play important roles in disease manifestation. Potential additional factors ad include genetic variants or non-genetic modifiers, such as gut microbiome or infections.

Infectious triggers are known to play a role in almost every autoimmune disease (AD) from rheumatic fever with *Streptococcus pyogenes*, to multiple sclerosis with Epstein-Barr virus (EBV), both by molecular mimicry (6–9). Kivity et al. described a bidirectional paradigm, referring to the phenomenon that while ADs are triggered by infections, on the other hand, patients with AD have a higher risk for acquiring infections or for the reactivation of latent ones (8). This phenomenon is well described in the case of systemic lupus erythematosus (SLE) and EBV (10, 11). Other common infectious agents have been described to trigger ADs as well: seroprevalence of anti-toxoplasma antibodies is reported to be elevated in anti-phospholipid syndrome, cryoglobulinemia, and rheumatoid arthritis (12). In the case of the anti-phospholipid syndrome, pathogenesis might be based on mimicry between *Toxoplasma gondii* antigens and phospholipids (13). Moreover – in part based on murine studies – human cytomegalovirus (CMV) is reported to contribute to autoimmunity in SLE in genetically predisposed subjects (14–16). Hence, not only infections but also genetic predisposition play a cardinal role in the susceptibility to developing autoimmune diseases. Studies in mice and humans have shown that a specific genetic constellation can lay grounds for an infection-triggered autoimmune disease (6, 8, 14, 15, 17).

Susceptibility to infections is a common clinical symptom among *CTLA4* mutation carriers, and as in the case of SLE, EBV infections or reactivations are common, leading to an increased risk of malignancy (3, 5, 18). However, whether infectious triggers are responsible for triggering the overt clinical phenotype in *CTLA4* mutation carriers has not been studied so far. Hence, we hypothesized that a yet unidentified infectious agent might trigger the disease onset in CTLA-4 insufficiency. A pathogen that is solely responsible for triggering the disease, must ideally have a prevalence of about 70% in the Western world, as this percentage corresponds to the disease penetrance observed in CTLA-4-families. Using this prevalence as a guide, we therefore studied the infection history and measured the humoral immune response in *CTLA4* mutation carriers against the following infectious agents: EBV (seroprevalence 90-95% (19, 20)), cytomegalovirus (CMV, seroprevalence 40-100% (21–23)), herpes simplex virus type 1 and 2 (HSV1/2; seroprevalence HSV1: 80-90%; HSV2: 10-25% (24, 25)), human parvovirus B19 (seroprevalence 40-80% (26, 27)), and *Toxoplasma gondii* (seroprevalence 50-77% (28, 29)). Our results show that the investigated pathogens have unlikely triggered the disease onset in CTLA-4 insufficient individuals and are not correlated with disease severity.

## Materials and methods

### Subjects and sample handling

*CTLA4* mutation carrier individuals (n=13) recruited in this study attend the outpatient clinic of the participating centers (Czech Republic: St Anne´s University Hospital in Brno; Germany: University of Freiburg, University of Heidelberg; University of Regensburg; Greece: Aghia Sophia Children’s Hospital, Athens). The participating individuals donated blood samples after signing an informed written consent under local ethics board–approved protocols Serum from total blood was separated and stored after sterile aliquoting at -80°C or in liquid nitrogen.

The Ethics Committee of the University of Freiburg has approved this research (protocol No. 466/18).

### Detection of IgG levels against selected pathogens

Seroprevalence was evaluated by detecting specific IgG against EBV, CMV, Parvovirus B19, HSV 1/2 and *Toxoplasma gondii* in serum samples collected from affected *CTLA4* mutation carriers and unaffected *CTLA4* mutation carriers who do not receive IgG replacement therapy.

The measurements for the selected pathogens were performed using the following reagents: Architect^®^ EBV EBNA-1 IgG, EBV VCA IgG (Abbott GmbH, Wiesbaden, Germany), LIAISON^®^ Biotrin Parvovirus B19 IgG, CMV IgG II (DiaSorin LIAISON, Saluggia, Italy), ELISA classic Herpes Simplex Virus 1 and 2 IgG (Serion, Würzburg, Deutschland) and LIAISON^®^ Toxoplasma gondii chemiluminescence immunoassay (CLIA). The assays were performed according to the manufacturer’s recommendations. To note: EBV VCA IgG was analyzed only when EBNA1 IgG was negative. The details of the used tests are summarized in **Table S3**.

### Fc gamma receptor activation assays (FcγR activation assays)

FcγRIIIA (CD16) activation was measured by a cell-based assay as previously described (30). The assay detects antigen–antibody interactions, which initiate an FcγR activation cascade on mouse BW-5147 reporter cells, eventually leading to IL-2 secretion. Briefly, 1x105 P3HR1 cells (EBV positive human Burkitt lymphoma cell line) were plated in 50 μL RPMI medium and incubated with 50 μL diluted serum sample (1:25, 1:125, and 1:625) for 1 hour at 37°C in a U-bottom-plate. A positive and a negative control (serum pool from 4 healthy, EBV seropositive or negative individuals, respectively) were always run in parallel. Following incubation, 2x105 FcγRIIIA (CD16) receptor-expressing BW-5147 reporter cells were added to the wells containing P3HR1 cells and cultured at 37°C overnight. After incubation, the cell-plates were frozen at -80°C for 15 minutes, and the supernatants were collected for IL2-enzyme-linked immunosorbent assay (ELISA). Briefly, ELISA plates were coated with purified rat anti-mouse IL-2 (BD-Pharmingen) diluted 1: 500 in 0.1M Na2HPO4 at 4°C. The plates were blocked with a blocking buffer (phosphate buffered saline (PBS) without Ca and Mg with 10% fetal calf serum (FCS) for 30 minutes at 37°C. Supernatants of the cell-plates described above were added to the ELISA plates and incubated for 1 hour at 37°C. Following three washing steps, the plates were incubated with Biotin rat anti-mouse IL-2 (Cat# 554426 BD-Pharmingen) diluted 1: 500 in the blocking buffer with 0.05% Tween 20 for 1 hour at 37°C followed by incubation with streptavidin-POD conjugate (Biozol) diluted 1:1000 in the blocking buffer with 0.05% Tween 20 for 30 minutes at room temperature and TMB substrate (Thermo Fisher Cat #34029). The reaction was stopped by adding 1 M H_2_SO_4_ and the optical density (OD) at 450 nm for each three-fold, serially diluted sample was measured. Activation levels were quantified by calculating the area under the curve (AUC) and expressed as activation index, which was obtained by normalizing the calculated AUC of each sample to the AUC value of the positive control.

### Statistical analysis

Statistical analysis was performed using GraphPad Prism 9.0.0 and R 4.1.1. For comparing odds ratios, two-sided Fisher’s exact test with Baptista-Pike method with 95% confidence intervals was used. For comparing results of the FcγRIIIA activation assay, an unpaired two-tailed t-test was used. The *p* values <0.05 were considered significant.

## Results

### Patient characteristics

We divided our cohort into two groups: (1) affected *CTLA4* mutation carriers (n=6) with autoimmune manifestations and (2) unaffected *CTLA4* mutation carriers (n=7). The affected *CTLA4* mutation carriers were defined as patients who suffer at the time of presentation or had suffered in the past from mild to severe symptoms caused by CTLA-4 insufficiency. None of the included individuals were on IgG-RT at the time of sample collection. The unaffected *CTLA4* mutation carriers included individuals who had no symptoms at the time of sample collection, as previously reported (3).

In total, we analyzed thirteen *CTLA4* mutation carriers coming from ten different families. Six of them were classified as affected (two severely, four mildly), and seven as unaffected. The severity of the clinical symptoms was evaluated by the treating physician and the recently published CHAI Morbidity Score (31). The CHAI Morbidity Score was developed for the ABACHAI clinical trial, however, in this current study, we utilized the published score to classify participating patients. The CHAI Score takes the main organ involvements of the CTLA-4 disease as its basis. In the current setting, we have calculated a cumulative score – for each organ we have taken retrospective data from the most severe disease state and calculated the score. Missing datasets were subtracted from the achievable total score. Lastly the percentage of achieved (out of total) was calculated. Patients below 10% were classified as unaffected, and a score of ≥10% lead to an affected classification. Between affected patients, patients with a score <20% were classified as mildly affected. The detailed breakdown of the scoring can be found in **Table S4**, further clinical datasets are summarized in **Table 1**. The male-to-female ratio was four to nine. The median age at the time of serum collection was 51 years (range 14.09-88.41). The included patients had missense (c.208C>T, p.R70W; c.511A>C, p.S171R; c.407C>T, p.P136L; c.373G>A, p.G125R; c.416A>G, pY139C), frameshift (c.402_415del, p.M123IfsTer15; c.165_190dup, p.G64AfsTer17), stop gained (c.105C>A; p.C35*) and splice site (c.109+1G>T) mutations. Additionally, the CTLA-4 transendocytosis assay has been performed – and had turned out defective – for all of the listed mutations for at least one index case (partially unpublished data), or the mutations have been reported previously in the literature (1, 3).

**Table 1:** Patient characteristics.

ID	Family	Status	Country	*CTLA4* mutation	Transendocytosis	Gender	Age at sample collection (years)	Patient code in case of previouspublication
P1	F1	Unaffected	Germany	c.105C>A; p.C35*	Schubert et al. (1) reduced	F	88	A.I.2 (1)
P2	F1	Unaffected	Germany	c.105C>A; p.C35*	Schubert et al. (1) reduced	M	50	A.II.10 (1)
P3	F1	Unaffected	Germany	c.105C>A; p.C35*	Schubert et al. (1) reduced	F	61	A.II.3 (1)
P4	F2	Affected (Mild)	Germany	c.109+1G>T	Schubert et al. (1) reduced	F	60	B.II.1 (1)
P5	F2	Unaffected	Germany	c.109+1G>T	Schubert et al. (1) reduced	F	17	B.III.3 (1)
P6	F3	Unaffected	Greece	c.208C>T; p.R70W	Schubert et al. (1) reduced	F	14	C.II.4 (1)
P7	F4	Affected (Mild)	Germany	c.511A>C, p.S171R	Performed, Unpublished reduced	F	46	Unpublished
P8	F4	Affected	Germany	c.511A>C, p.S171R	Performed, Unpublished reduced	M	20	Unpublished
P9	F5	Affected	Germany	c.407C>T; p.P136L	Performed, Unpublished reduced	F	52	H.I.2 (3)
P10	F6	Unaffected	Germany	c.373G>A; p.G125R	Not performed	F	49	J.1.2 (3)
P11	F7	Unaffected	Czech Republic	c.402_415del; p.M134IfsTer15	Performed, unpublished reduced	M	57	KK.I.1 (3)
P12	F8	Affected	Germany	c.165_190 dupp. p.G64AfsTer17	Rojas-Restrepo et al. (32)reduced	M	70	Unpublished
P13	F9	Affected (Mild)	Germany	c.416A>G, p.Y139C	Performed, unpublished reduced	F	33	Unpublished

*refers to a stop codon/termination as of http://varnomen.hgvs.org/recommendations/protein/variant/frameshift/.

### Seroprevalence

To investigate the influence of the infection history as disease trigger, we compared affected and unaffected *CTLA4* mutation carriers’ seroprevalence for EBV (EBNA-1) IgG, CMV IgG, parvovirus B19 IgG, HSV-1/2 IgG and *Toxoplasma gondii.*

**Figure 1:**
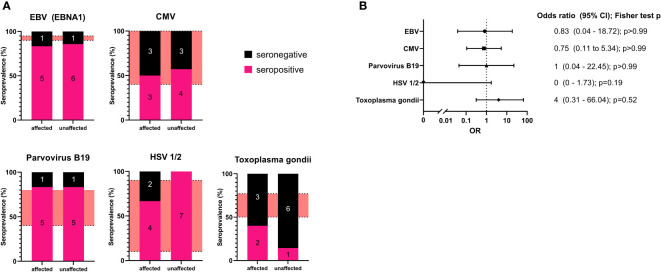
**(A)**: Seroprevalence analysis for the examined pathogens in affected and unaffected *CTLA4* mutation carriers. The number of individuals is included in each section of the bar chart as an annotation. The red areas refer to seroprevalence reported in the literature. **(B)** Forest plot of the calculated odds ratio for being affected in case of seropositivity for each examined pathogen. Odds ratios and confidence intervals are shown on the right with calculated p values (two-sided Fisher’s exact test with Baptista-Pike method with 95% confidence intervals).

The seropositivity for affected or unaffected *CTLA4* mutation carriers for each pathogen were: EBV (EBNA1): 5 of 6 (83%) and 6 of 7 (86%); CMV IgG: 3 of 6 (50%) and 4 of 7 (57%); parvovirus B19: 5 of 6 (83%) and 5 of 6 (83%); for HSV-1/2: 4 of 6 (67%) and 7 of 7 (100%); and for *Toxoplasma gondii*: 2 of 5 (40%) and 1 of 6 (14%), respectively. (**Figure 1A**; **Table 2**).

**Table 2:** Experimental results. In the table pos: positive, neg: negative, eqv: borderline result.

ID	HSV 1/2 (U/ml)	HSV 1/2interpretation	CMV (AU/ml)	CMV interpretation	EBV EBNA1 (S/Co)	EBNA1 interpretation	EBV VCA-IgG	EBV VCA interpretation	Parvovirus B19 (Index)	Parvovirus B19 interpretation	Toxoplasma gondii IgG (IU/ml)	Toxoplasma gondii interpretation	EBV-FCgRIII activation index
P1	1005.9	pos	92.8	pos	2.4	pos			0.1	neg	21.1	pos	0.578233103
P2	735.5	pos	<5	neg	13.71	pos			46	pos	<3.00	neg	1.113358169
P3	1051.4	pos	97.5	pos	21.06	pos			6.1	pos	<3.00	neg	1.125864822
P4	1649.7	pos	>180	pos	15.13	pos			31	pos	16.6	pos	1.446514103
P5	2993	pos	79.2	pos	0.28	neg	63.65	pos	15	pos	<3.00	neg	0.936402342
P6	2136.1	pos	<5	neg	17.34	pos			0.94	eqv	<3.00	neg	0.791910591
P7	183.98	pos	<5	neg	9.61	pos			0.33	neg	14.4	pos	0.928419372
P8	2.58	neg	<5	neg	0.01	neg	0.01	neg	2	pos	<3.00	neg	0.408195849
P9	1567.8	pos	<5	neg	9.9	pos			41	pos	NA	NA	1.600851517
P10	1616	pos	<5	neg	21.35	pos			45	pos	<3.00	neg	1.480042576
P11	703.49	pos	180	pos	18.08	pos			2.1	pos	<3.00	neg	NA
P12	25	pos	>180	pos	23.39	pos			34	pos	<3	neg	1.005333952
P13	<0.5	neg	115	pos	22	pos			37	pos	<3	neg	0.984230056

NA, not available.

Our results show that affected *CTLA4* mutation carriers did not present with higher seroprevalence for the examined pathogens compared to unaffected mutation carriers. However, in the case of *Toxoplasma gondii*, we found all but one unaffected *CTLA4* mutation carriers to be seronegative but also only two of five affected individuals to be positive. The two more severely affected patients (P8, P12) both tested negative for *Toxoplasma gondii.*

Additionally, we have determined the odds ratio (OR) for being affected by CTLA-4 insufficiency with relation to seropositivity (two-sided Fisher’s exact test with Baptista-Pike method with 95% confidence intervals). This analysis did not reveal any correlation for most of the pathogens. For *Toxoplasma gondii*, we observed an increased OR for seropositivity; however, statistical significance was not reached (**Figure 1B**).

### Fc gamma receptor activation assays (FcγR assay) for EBV

EBV is a very common viral agent in CTLA-4 insufficient patients (5, 18) and showed one of the highest seroprevalence in our cohort. To evaluate a possible IgG-dysfunction in B cells from CTLA-4-insufficient patients, we analyzed – by means of a cell-based reporter assay (30) – the ability of EBV-specific IgG opsonizing infected cells to activate FcγRIII/CD16A, a prototypical activating FcγR expressed most notably by NK cells and monocyte-derived macrophages. This assay was instrumental in identifying an augmented activation potential of EBV-specific IgG in patients with a complete hereditary deficiency of FcγRIIIA/CD16A suffering from persistent EBV infection (33).

**Figure 2:**
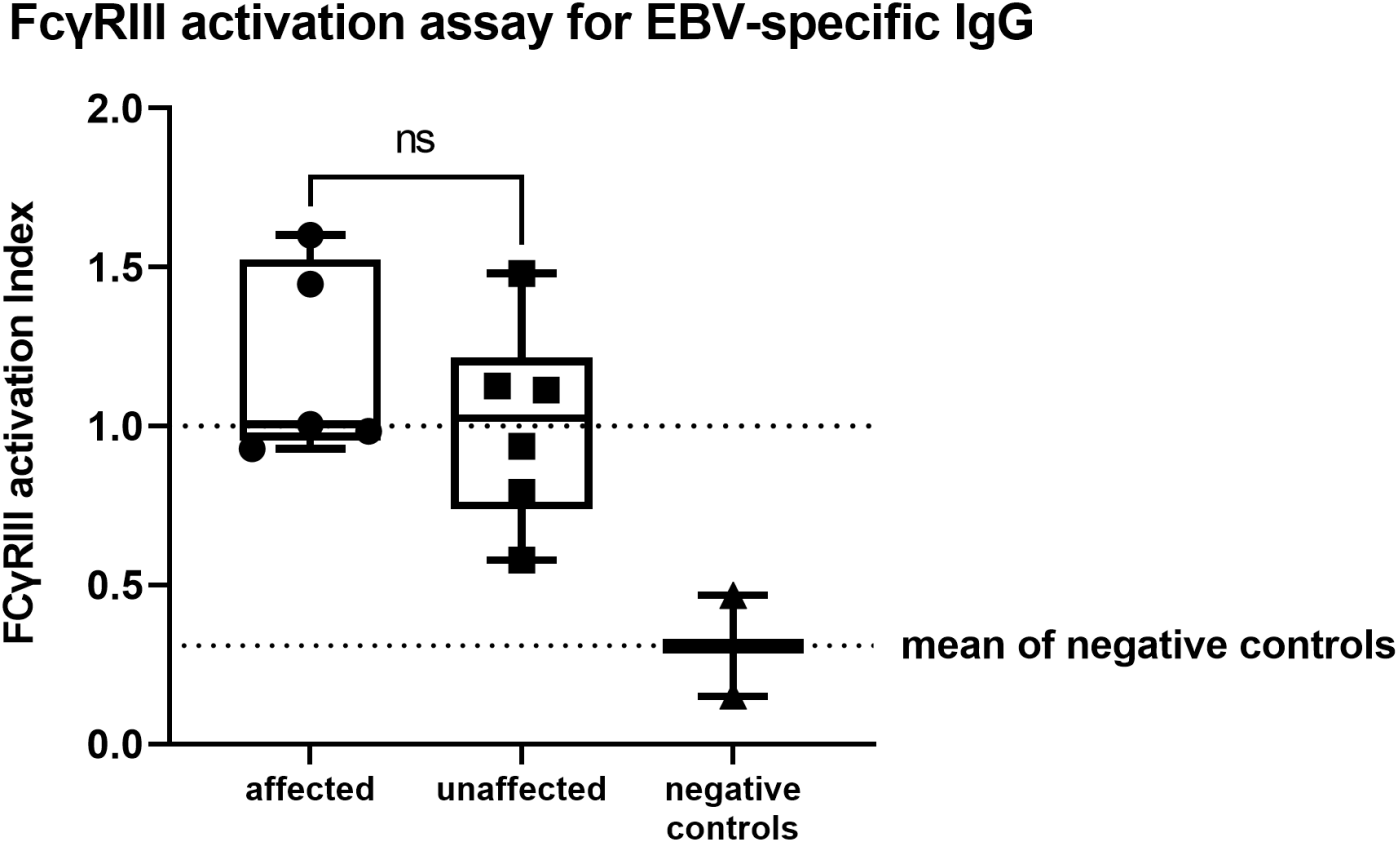
FcγRIII activation assay for EBV-specific IgG showing no significant differences between affected or unaffected *CTLA4* mutation carriers (unpaired two-tailed t-test; p=0.3477). Activation levels were quantified by calculating the area under the curve (AUC) and expressed as activation index, which was obtained by normalizing the calculated AUC of each sample to the AUC value of the positive controls. Positive and negative controls represent a serum pool from four healthy, EBV seropositive, or negative individuals. ns, not significant.

All of the affected patients showed FcγRIII activation levels similar to or slightly higher than the positive controls (see *Methods*). Patient 8 resulted EBV-seronegative and was therefore excluded from this analysis. In the unaffected group, we detected a reduced activity for P1, who was completely unaffected at the time of evaluation, and a slightly reduced activity for P6. However, all patients in this group showed FcγRIII activation levels higher than the negative control. Overall, the ability of EBV-specific IgG to activate FcγRIII did not significantly differ between affected and unaffected *CTLA4* mutation carriers (unpaired two-tailed t-test; p=0.3405, **Figure 2**; **Table 2**).

Increased susceptibility to infections was not reported, despite the observed asymptomatic hypogammaglobulinemia, which might correlate to the IgG-dysfunction. P5, who tested negative for EBNA-1 but positive for VCA-IgG, showed FcγRIII-activation similar to the positive control. In line with this dataset, around the time of evaluation ( ± 6 months), EBV-replication was detected in the patient’s serum (PCR weakly positive).

## Discussion

In the present study, we evaluated the seroprevalence of common infectious agents as a potential disease modifier factor in the context of CTLA-4 insufficiency, as this autosomal dominant disease presents with reduced penetrance. Our results show no obvious association of the infection history for the pathogens mentioned above with the onset or severity of CTLA-4 insufficiency.

Seroprevalence for the examined pathogens was not significantly different between affected and unaffected *CTLA4* mutation carriers. Additionally, the observed seropositivity of both groups was within the range of that reported for healthy reference populations. According to our results, one may speculate that a previous infection with *Toxoplasma gondii* might contribute to disease manifestation. In fact, we observed a higher (but statistically insignificant) Toxoplasma gondii seroprevalence in the affected group compared to the unaffected one. However, the finding that two more severely affected patients (P8 and P12) were tested seronegative (**Figure S1**) does not support this hypothesis. As we show that previous exposures to EBV, CMV, HSV1/2, or parvovirus B19 are not obviously associated with an overt CTLA-4 phenotype, other factors are required to induce clinical disease manifestation.

EBV reactivation is common amongst *CTLA4* mutation carriers, regardless of their clinical status, and asymptomatic viremia is a common phenomenon in our patients (3, 5, 18). Mechanisms underlying the susceptibility to EBV have been suggested, such as dysfunction in T and NK cells: Hoshino et al. showed normally functioning EBV-specific CD8+ T cells in affected and unaffected mutation carriers, suggesting that the infection itself might not trigger disease onset directly (18). Impaired NK cell function has also been reported in CTLA-4 insufficient patients, which might contribute to the frequent EBV viremia, however, its relevance as a disease trigger is yet to be investigated (34).

On the other hand, we hypothesized that a dysfunctional or excessive production and function of IgG against EBV could be an important element underlying the susceptibility to EBV in CTLA-4 insufficiency. Our results show, however, that both affected and unaffected *CTLA4* mutation carriers, who do not need immunoglobulin replacement therapy, can produce normally functioning antibodies against EBV, at least with regard to FcγRIII activation. The one unaffected patient with impaired FcγR activation in our cohort (P1) presented with hypogammaglobulinemia at the time of evaluation, which might be in line with the impaired IgG function but had no history of infection susceptibility. Taken together, dysfunctional IgG production against EBV is unlikely to promote disease onset or be the cause of EBV-susceptibility. Even if an EBV infection itself may not promote disease onset, it is important to mention that EBV (re)activation can dramatically change the disease course of CTLA-4 insufficiency. More than half of the reported cancers in CTLA-4 patients are EBV-associated, which observation (including additional virus-related cancer cases) leads to the speculation that the CTLA-4 patients fail at controlling (i.a. oncogenic) viruses (5).

Our study was limited by the fact that we could only examine *CTLA4* mutation carriers without IgG replacement therapy. This serological approach leads to excluding the majority of CTLA-4 insufficiency patients, as around 76% of the CTLA-4 patients receive IgG-replacement (4). The use of serological tests presents an additional limitation, as serology does not provide information about the time of the infection, therefore time-related correlations between disease-onset and the infections cannot be made.

Our results altogether show that it is unlikely that a previous exposition to the examined pathogens would lead to disease onset in CTLA-4 insufficiency. Our conclusion is supported by the observation of very young infants with severely symptomatic CTLA-4 insufficiency manifesting as early as 20 months of age (35), without a significant infection history. We have also followed a family in which the newborn *CTLA4*-mutated child developed severe recalcitrant arthritis at the age four months, having had no obvious infection other than the live rotavirus immunization. Moreover, as the prevalence of rotaviruses is close to 100% (36, 37), this does not explain the reduced penetrance of 70% seen in our CTLA-4 cohort. The case report on this child can be viewed below. Other vaccines are also very unlikely to trigger the disease, as our CTLA-4 patients had received their regular childhood vaccinations without any complications, including the mRNA Covid-19 vaccine, to which some of our CTLA-4 patients mounted a good response (Ochoa et al., manuscript under preparation).

**Case report:** This patient is a currently 1.5-year-old female. She was born to non-consanguineous parents. While her father is healthy, her mother has had a history of autoimmune thyroiditis and partial alopecia since her teenage years. Pregnancy and perinatal history were uneventful. Vaccinations were introduced at eight weeks of age according to national STIKO recommendations (38) and tolerated well, including tetanus, diphtheria, pertussis, Haemophilus influenzae B, poliomyelitis, hepatitis B, and pneumococcus, as well as the attenuated live rotavirus vaccine. She remained clinically asymptomatic until the age of four months when she presented with sudden onset of a non-infectious oligoarthritis with painful swellings of single toes and fingers. Until six months of age, arthritis worsened to polyarthritis, which bilaterally involved wrists, fingers and toes, as well as the left shoulder, right hip, knee, and ankle. Laboratory assessment showed leukocytosis (15,72 G/l), thrombocytosis (801 G/l) as well as elevation of CRP (40 mg/l), soluble IL2-receptor (1928 kU/l) and IgG (1840 mg/dl). Anti-nuclear antibodies (ANA) and an HLA-B27 screen were negative. The patient was started on corticosteroids and methotrexate, but clinical symptoms and laboratory signs of inflammation persisted. Additionally, pathological generalized lymph node enlargements occurred from seven months onwards. The spleen was not enlarged. At ten months of life, an exome-based gene panel for inborn errors of immune regulation was initiated and identified a previously reported pathogenic variant in *CTLA4* (c.416A>G; p.Tyr139Cys). Family screening identified maternal inheritance. In the context of the diagnosis of CTLA-4 insufficiency, the immune phenotype was assessed, and identified an expansion of CD8 cells with a senescent phenotype (70% of CD8 cells expressing CD57). Otherwise, the total numbers of CD3, CD4, CD8, CD19, CD16/56 as. well as naïve CD4 and FOXP3+ Treg cells were normal for age. Vaccine responses (tetanus) were detectable. Targeted therapy with abatacept (50 mg/week s.c.) was initiated at 11 months of age and increased to 75 mg at 13 months, but the patient’s extended oligoarthritis was not responsive to this treatment. Significant improvement was only seen after an additional course of rituximab (4x375 mg/m2/week) from 14 months onward. Given the early onset and aggressive course of CTLA-4 insufficiency associated oligoarthritis, the patient is currently scheduled for allogeneic hematopoietic stem cell transplantation from a matched unrelated donor (MUD). The live-attenuated MMRV vaccination was postponed and not given, as she was under immunomodulatory treatment at age 12 months.

Similar to other inborn errors of immunity with reduced penetrance, no disease modifiers in CTLA-4 insufficiency have yet been identified, including gender, somatic mutations, or the HLA locus (39). Our study shows that it is unlikely that a single pathogen might be able to trigger disease-onset – it is likely that triggering of the ouvert disease needs the interaction of several factors, such as genetic variants (40), or the gut microbiota. Current studies aiming to analyze genetic and epigenetic disease-modifying factors are running in the author’s research group. Infections during childhood can be implicated in the development of autoimmune diseases in adulthood (8). Therefore, prospective monitoring of mutation-carrying individuals may shed additional light on the role of infectious agents in CTLA-4 insufficiency.

## Data availability statement

The original contributions presented in the study are included in the article/**Supplementary Material**. Further inquiries can be directed to the corresponding author.

## Ethics statement

The studies involving human participants were reviewed and approved by The Ethics Committee of the University of Freiburg (protocol No. 466/18). The patients/participants provided their written informed consent to participate in this study.

## Author contributions

BG, LG-D, and NM performed study design and supervision. BG, NM, and MKr wrote the first draft of the manuscript. BG, NM, and MKr performed clinical data analysis. VF, NM, MKr, SP-C, and JK did experimental data analysis. VF, NM, and JK produced experimental data. BG, NM, MKr, JL, H-ML, DW, CS, AH, and MKa provided and collected clinical data. BG, HH, and GH provided supervision and funding. All authors have reviewed, contributed to and approved the final version of the manuscript.

## Funding

This work was supported by the Deutsche Forsch-ungsgemeinschaft (DFG) IMPATH SFB1160/2_B5. BG receives support by the Deutsche Forschungsgemeinschaft (DFG) SFB1160/2_B5, under Germany’s Excellence Strategy (CIBSS – EXC-2189 – Project ID 390939984, and RESIST – EXC 2155 – Project ID 390874280); by the E-rare program of the EU, managed by the DFG, grant code GR1617/14-1/iPAD; and by the German Federal Ministry of Education and Research (BMBF) through a grant to the German Auto-Immunity Network (GAIN), grant code 01GM1910A. HH is funded by the Deutsche Forschungsgemeinschaft (DFG) through HE2526/9-1. MK is supported by the Deutsche Forschungsgemeinschaft (DFG) SFB1160_2 as clinician scientist associated to IMM-PACT-Program, Faculty of Medicine, University of Freiburg, Freiburg, Germany. We acknowledge support by the Open Access Publication Fund of the University of Freiburg.

## Acknowledgments

We thank all participant patients and families. We thank Nathalie Göppert for the excellent technical assistance. Some samples for this project were obtained from the CCI-Biobank, a partner biobank of the University Medical Center Freiburg and Medical Faculty “Center for Biobanking – FREEZE”.

## Conflict of interest

The authors declare that the research was conducted in the absence of any commercial or financial relationships that could be construed as a potential conflict of interest.

## Publisher’s note

All claims expressed in this article are solely those of the authors and do not necessarily represent those of their affiliated organizations, or those of the publisher, the editors and the reviewers. Any product that may be evaluated in this article, or claim that may be made by its manufacturer, is not guaranteed or endorsed by the publisher.

## Abbreviations

ANA, Anti-nuclear antibodies; AUC, area under the curve; CLIA, Chemiluminescence Immunoassay; CMV, Cytomegalovirus; COPD, chronic obstructive pulmonary disease; CTLA-4, Cytotoxic-T-lymphocyte-antigen-4; EBV, Epstein-Barr virus; FCS, fetal calf serum; HSV, herpes simplex virus; OR, odds ratio; PBS, phosphate buffered saline; SLE, systemic lupus erythematosus; STIKO, Standing Committee on Vaccination (Ständige Impfkommission).
