# Corrigendum: Do common infections trigger disease-onset or -severity in CTLA-4 insufficiency?

**DOI:** 10.3389/fimmu.2023.1226814

**Published:** 2023-06-02

**Authors:** Máté Krausz, Noriko Mitsuiki, Valeria Falcone, Johanna Komp, Sara Posadas-Cantera, Hanns-Martin Lorenz, Jiri Litzman, Daniel Wolff, Maria Kanariou, Anita Heinkele, Carsten Speckmann, Georg Häcker, Hartmut Hengel, Laura Gámez-Díaz, Bodo Grimbacher

**Affiliations:** ^1^Institute for Immunodeficiency, Medical Center – University of Freiburg, Faculty of Medicine, University of Freiburg, Freiburg, Germany; ^2^Center for Chronic Immunodeficiency (CCI), Medical Center – University of Freiburg, Faculty of Medicine, University of Freiburg, Freiburg, Germany; ^3^Department of Rheumatology and Clinical Immunology, Medical Center - University of Freiburg, Faculty of Medicine, University of Freiburg, Freiburg, Germany; ^4^Faculty of Biology, University of Freiburg, Freiburg, Germany; ^5^Institute of Virology, University Medical Center and Faculty of Medicine, University of Freiburg, Freiburg, Germany; ^6^Institute of Medical Microbiology and Hygiene, Medical Center - University of Freiburg, Faculty of Medicine, University of Freiburg, Freiburg, Germany; ^7^Division of Rheumatology, Department of Internal Medicine V, University of Heidelberg, Heidelberg, Germany; ^8^Department of Clinical Immunology and Allergology, St. Anne’s University Hospital in Brno and Medical Faculty, Masaryk University, Brno, Czechia; ^9^Department of Internal Medicine III, University Hospital Regensburg, Regensburg, Germany; ^10^Department of Immunology and Histocompatibility, Centre for Primary Immunodeficiencies, “Aghia Sophia” Children’s Hospital, Athens, Greece; ^11^Center for Pediatric Rheumatology, Olgahospital, Stuttgart, Germany; ^12^Department of Pediatric Hematology and Oncology, Center for Pediatrics and Adolescent Medicine, Faculty of Medicine, University of Freiburg, Freiburg, Germany; ^13^DZIF – German Center for Infection Research, Satellite Center Freiburg, Freiburg, Germany; ^14^CIBSS – Centre for Integrative Biological Signalling Studies, University of Freiburg, Freiburg, Germany; ^15^RESIST – Cluster of Excellence 2155 to Hannover Medical School, Satellite Center Freiburg, Freiburg, Germany

**Keywords:** cytotoxic T-lymphocyte antigen 4 (CTLA-4), immunodeficencies, immune dysregulation, inborn errors of immunity (IEI), disease modifiers

In the published article, there was an error in the Funding statement. The following funding information is missing: “We acknowledge support by the Open Access Publication Fund of the University of Freiburg.” The correct Funding statement appears below.

