# Supplementary material for: Do common infections trigger disease-onset or -severity in CTLA-4 insufficiency?
Source: Front Immunol. 2022 Nov 2;13:1011646. doi: 10.3389/fimmu.2022.1011646 (PMC9667032; doi:10.3389/fimmu.2022.1011646)
Supplement: Supplementary file 1 [file DataSheet_1.docx]

Supplementary Material

# Supplementary figures


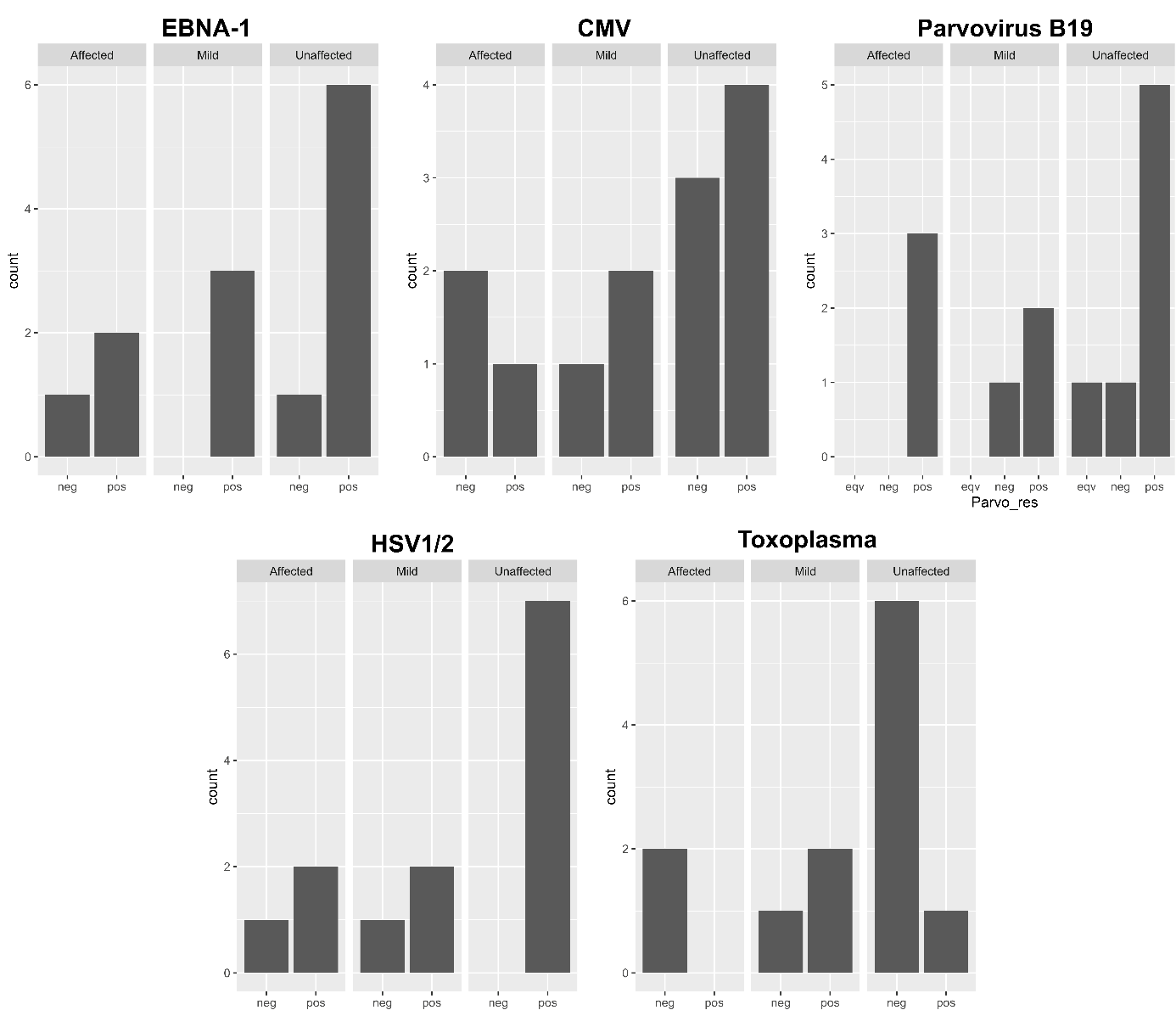


**Figure S1:** Detailed breakdown of the serological results for the examined pathogens in affected, mildly affected, and unaffected *CTLA4* mutation carriers.

Table S1: Clinical phenotype of the patients. In the table, 0 = absent, 1 = present, NA = data not available

| **ID** | **Relevant infections in the patient history, increased susceptibility to infections or major infections** | **Infections detail** | **Autoimmune disease** | **Autoimmune detail** | **Lymphoproliferation** | **Lymphoproliferation_detail** | **Other** | **Other detail** | **Hypo/Dysgamma** | **IgG replacement** | **Immunosuppression therapy** |
| --- | --- | --- | --- | --- | --- | --- | --- | --- | --- | --- | --- |
| P1 | 0 | 0 | 0 | 0 | 0 | 0 | 1 | gastric cancer | 1 | 0 | 0 |
| P2 | 0 | 0 | 0 | 0 | 0 | 0 | 0 | diarrhea (no further details) | 0 | 0 | 0 |
| P3 | 0 | 0 | 1 | psoriasis | 0 | 0 | 0 | diarrhea | 0 | 0 | 0 |
| P4 | 1 | bacterial fasciitis and sepsis at age 59, soor | 1 | autoimmune enteropathy, mild (recent endoscopy did not show histological evidence for duodenitis or sprue) | 0 | 0 | 1 | eczematous skin disease | 1 (IgG2 subclass) | 0 | 0 |
| P5 | 1 | often viral infections, but without increased susceptibility | 0 | at the time of analysis no, however, age 22: Anti-TPO and Thyreoglobulin-antibody positive | 0 | 0 | 0 | 0 | 1 | 0 | 0 |
| P6 | 0 | 0 | 0 | 0 | 0 | 0 | 1 | history of growth delay (<3rd percentile, height, weight), intestinal symptoms in late infancy (but no confirmed inflammatory bowel disease) | 0 | 0 | 0 |
| P7 | 0 | 0 | 1 | ulcerative colitis, protracted diarrhea, atopic dermatitis | NA | NA | 0 |  | 0 | 0 | 0 |
| P8 | 1 | recurrent colds, at age 20 otitis media | 1 | Crohn´s disease, protracted diarrhea, Hashimoto, atopic dermatitis, alopecia | 1 | lymphatic hyperplasia in terminal ileum | 0 | 0 | 1 | 0 | previously corticosteroids, azathioprine |
| P9 | 0 | 0 | 1 | type 1 diabetes, vitiligo, stiff-person syndrome), pernicious anemia (type A gastritis), colitis | 0 | NA | 1 | asthma, pancreas insufficiency, bronchiectasis | NA | 0 (occasionally for the stiff-person syndrome, but not around the time of sample collection for this study) | NA |
| P10 | 0 | 0 | 0 |  | 0 | 0 | 0 | 0 | 0 | 0 | 0 |
| P11 | 0 | 0 | 1 | pernicious anemia | 0 | 0 | 0 | 0 | 0 | 0 | 0 |
| P12 | 0 | 0 | 1 | Hashimoto, autoimmune enteropathy, nephropathy of unknown origin | 1 | intraepithelial lymphocytosis Marsh 3c | 1 | COPD, macular degeneration | 0 | 0 | budesonide, abatacept |
| P13 | 1 | as a child recurrent sinusitis, otitis, tonsillitis, COVID-19 | 1 | Hashimoto, alopecia, skin eczema | 0 |  | 1 | endometriosis (no records) | 1 (IgG2 subclass decreased transiently) | 0 | abatacept |

Table S2: Immunological laboratory datasets of the patients. NA = not available

| **ID** | **Age for Lab Data** | **IgG (g/L)** | **IgA (g/L)** | **IgM (g/L)** | **Lymphocyte (/µl)** | **CD19+ (/µl)** | **CD19+ of Lymphocytes (%)** | **switched memory B-cells out of B-cells (%)** | **Tetanus**  **vaccine response** | **Diphtheria**  **vaccine response** | **Pneumococcus vaccine response** |
| --- | --- | --- | --- | --- | --- | --- | --- | --- | --- | --- | --- |
| P1 | 73 | 4,22 | 0,25 | 0,44 | NA | NA | NA | NA | NA | NA | NA |
| P2 | 37 | 8,98 | 1,92 | 1,3 | NA | NA | NA | NA | NA | NA | NA |
| P3 | 63 | at age 61 within normal range, at age 63 no values available | | | 1401 | 113 | 8,1 | 7,84 | NA | NA | NA |
| P4 | 60 | 11,6 | 1,02 | 1,11 | 1408 | 122 | 8,7 | NA | absent | absent | NA |
| P5 | 17 | 14,7 | 0,08 | 0,68 | 1001 | 187 | 18,7 | NA | NA | NA | NA |
| P6 | 14 | 15 | 2,32 | 2,04 | 1820 | 344 | 18,9 | NA | NA | NA | NA |
| P7 | 46 | 15,2 | 1,29 | 0,66 | 1202 | 131 | 10,9 | 10,69 | positive (at age 45) | positive (at age 45) | NA |
| P8 | 20 | 6,71 | 0,38 | 0,44 | 406 | 85 | 21 | 1,31 | positive (at age 19) | positive (at age 19) | NA |
| P9 | NA | NA | NA | NA | NA |  | NA | NA | NA | NA | NA |
| P10 | NA | NA | NA | NA | NA | NA | NA | NA | NA | NA | NA |
| P11 | 58 | 8,6 | 2 | 1,2 | 1670 | 80 | 5 | 21,9 | positive | positive | positive |
| P12 | 70 | 8,19 | 2,15 | 0,27 | 1671 | 150 | 9 | 38,28 | NA | NA | positive |
| P13 | 33 | 11,7 | 2,45 | 0,8 | 1331 | 182 | 13,6 | 10,53 | NA | NA | positive |

**Table S3**: Summary of the used serological tests

| **Test** | **Company** | **Kit** | **Test principle** | **Units** | **negative** | **borderline** | **positive** |
| --- | --- | --- | --- | --- | --- | --- | --- |
| EBV EBNA 1 | Abbott (Wiesbaden, Germany) | 3P67 Architect EBV EBNA-1 IgG | Chemiluminescence Microparticle Immunoassay (CMIA). | S/CO | <0,5 | 0,5 to <1 | > 1 |
| EBV VCA | Abbott (Wiesbaden, Germany) | 3P65 Architect EBV VCA IgG | Chemiluminescence Microparticle Immunoassay (CMIA). | S/CO | < 0,75 | 0,75 to <1 | > 1 |
| Parvo IgG | Dia Sorin (Saluggia, Italy) | LIAISON® Biotrin Parvovirus B19 IgG ([REF] 317000) | Chemiluminescence Microparticle Immunoassay (CMIA). | Index | < 0,9 | 0,9 to <1,1 | >1,1 |
| CMV IgG | Dia Sorin (Saluggia, Italy) | LIAISON® CMV IgG II ([REF] 310745 | Chemiluminescence Microparticle Immunoassay (CMIA). | U/ml | <12 | 12-14 | >14 |
| HSV IgG | Serion (Würzburg, Deutschland) | ELISA classic Herpes Simplex Virus 1+2 Ig GESR105G | ELISA |  | <20 | 20-40 | >40 |
| Toxoplasma IgG | Dia Sorin (Saluggia, Italy) | LIAISON® Toxoplasma gondii IgG | Chemiluminescence Immunoassay (CLIA). | IU/ml | <7,2 | 7,2-8,8 | >8,8 |

**Table S4: CHAI Morbidity Score calculations for each patient.** The numbers in the table refer to the given score for each category based on the original publication (see main text). NA refers to missing datasets. 0 (NA) is used in cases, where no data was available – especially in clinically unaffected cases – but based on other clinical datasets and conditions, a score of 0 can be assumed (e.g. there was no indication for evaulation).

| **parameter** | **P1** | **P2** | **P3** | **P4** | **P5** | **P6** | **P7** | **P8** | **P9** | **P10** | **P11** | **P12** | **P13** |
| --- | --- | --- | --- | --- | --- | --- | --- | --- | --- | --- | --- | --- | --- |
| **Lung involvement (GLILD)** |  |  |  |  |  |  |  |  |  |  |  |  |  |
| DLCOc/VA | 0 (NA) | 0 (NA) | 0 (NA) | 1 | 0 | 0 (NA) | 0 (NA) | 0 (NA) | NA | 0 (NA) | 0 (NA) | 3 | 0 |
| GLILD-typical lesions affecting the lungs in CT | 0 (NA) | 0 (NA) | 0 (NA) | 0 | 0 (NA) | 0 (NA) | 0 | 0 | NA | 0 (NA) | 0 (NA) | 0 | 0 |
| **Gut involvement (enteropathy)** |  |  |  |  |  |  |  |  |  |  |  |  |  |
| stool frequency and quality within the last 24h | 1 | 1 | 0 | 1 | 0 | 0 | 2 | 1 | 3 | 0 (NA) | 0 (NA) | 3 | 0 |
| weight loss compared to baseline | NA | NA | 0 (NA) | 0 | 0 | 0 | 0 | 0 | NA | 0 (NA) | 0 (NA) | 3 | 0 |
| serum potassium | NA | NA | 0 | 0 | 0 | 0 (NA) | 0 | 0 | NA | 0 (NA) | 0 (NA) | 2 | 0 |
| **Cytopenia** |  |  |  |  |  |  |  |  |  |  |  |  |  |
| thrombocytopenia | 0 (NA) | 0 (NA) | 0 | 0 | 0 | 0 (NA) | 0 | 0 | 0 (NA) | 0 (NA) | 0 (NA) | 0 | 0 |
| anemia | 0 (NA) | 0 (NA) | 0 | 0 | 0 | 0 (NA) | 0 | 0 | 0 (NA) | 0 (NA) | 0 (NA) | 0 | 0 |
| **CNS involvement** |  |  |  |  |  |  |  |  |  |  |  |  |  |
| cerebral lesions in CT/MRI | 0 (NA) | 0 (NA) | 0 (NA) | 0 (NA) | 0 (NA) | 0 (NA) | 0 (NA) | 0 (NA) | NA | 0 (NA) | 0 (NA) | 0 (NA) | 0 (NA) |
| neurological impairment based on the NANO-Scale | 0 (NA) | 0 (NA) | 0 (NA) | 0 (NA) | 0 (NA) | 0 (NA) | 0 (NA) | 0 (NA) | NA | 0 (NA) | 0 (NA) | 0 | 0 |
| **Immune system** |  |  |  |  |  |  |  |  |  |  |  |  |  |
| absolute lymphocyte count | NA | NA | 0 | 0 | 1 | 0 | 0 | 3 | NA | NA | 0 | 0 | 0 |
| absolute CD4+ T cell count | NA | NA | 0 | 0 | 2 | 0 | 0 | 2 | NA | NA | NA | 0 | 0 |
| naïve (CD4+CD45RA+) T cell % of CD4+ | NA | NA | 0 | 1 | 1 | 0 | 0 | 0 | NA | NA | NA | 2 | 1 |
| CD4+ T cell activation (HLA-DR+ in CD4+) | NA | NA | 0 | 0 | 0 | 0 | 0 | 0 | NA | NA | NA | 0 | 0 |
| PD1 expression on CD8+ T cells | NA | NA | NA | NA | NA | NA | NA | NA | NA | NA | NA | NA | NA |
| switched memory (CD19/20+, IgM-, CD27+) B cell numbers in % of B cells | NA | NA | 0 | 0 | 0 | 0 | 0 | 3 | NA | NA | 0 | 0 | 0 |
| **Lymphoproloferation** |  |  |  |  |  |  |  |  |  |  |  |  |  |
| splenomegaly | 0 (NA) | 0 (NA) | 0 | 0 | 0 | 0 | 0 | 0 | NA | 0 (NA) | 0 (NA) | 0 | 0 |
| lymphadenopathy (give points corresponding the scoring chart) | 0 (NA) | 0 (NA) | 0 | 0 | 0 | 0 | 0 | 0 | NA | 0 (NA) | 0 (NA) | 0 | 0 |
| sIL2R (U/ml) | NA | NA | NA | 1 | 0 | NA | 2 | 3 | NA | 0 (NA) | 0 (NA) | 3 | 0 |
| **Skin involvement** |  |  |  |  |  |  |  |  |  |  |  |  |  |
| distribution | 0 (NA) | 0 (NA) | 1 | 1 | 0 | 0 | 1 | 1 | 1 | 0 (NA) | 0 (NA) | 0 | 3 |
| type of skin lesions | 0 (NA) | 0 (NA) | 1 | 1 | 0 | 0 | 1 | 1 | 1 | 0 (NA) | 0 (NA) | 0 | 2 |
| **total achievable** | 33 | 33 | 42 | 57 | 57 | 54 | 57 | 57 | 15 | 42 | 48 | 57 | 57 |
| **total reached** | 1 | 1 | 2 | 6 | 4 | 0 | 6 | 14 | 5 | 0 | 0 | 16 | 6 |
| **reached % of achievable** | 3% | 3% | 5% | 11% | 7% | 0% | 11% | 25% | 33% | 0% | 0% | 28% | 11% |
| **classification** | unaffected | unaffected | unaffected | affected (mild) | unaffected | unaffected | affected (mild) | affected | affected | unaffected | unaffected | affected | affected (mild) |
